# Therapeutic Nonsense Suppression Modalities: From Small Molecules to Nucleic Acid-Based Approaches

**DOI:** 10.3390/biomedicines12061284

**Published:** 2024-06-10

**Authors:** Pedro Morais, Rui Zhang, Yi-Tao Yu

**Affiliations:** 1 Drug Metabolism and Pharmacokinetics, Research and Development, Bayer Pharmaceuticals, 42113 Wuppertal, Germany; 2 Center for RNA Biology, Department of Biochemistry and Biophysics, University of Rochester Medical Center, 601 Elmwood Avenue, Rochester, NY 14642, USA; rzhang44@ur.rochester.edu

**Keywords:** nonsense suppression, nonsense-mediated mRNA decay, new modalities, premature stop codons, small molecules, mRNA, oligonucleotides, RNA editing, pseudouridylation, CRISPR

## Abstract

Nonsense mutations are genetic mutations that create premature termination codons (PTCs), leading to truncated, defective proteins in diseases such as cystic fibrosis, neurofibromatosis type 1, Dravet syndrome, Hurler syndrome, Beta thalassemia, inherited bone marrow failure syndromes, Duchenne muscular dystrophy, and even cancer. These mutations can also trigger a cellular surveillance mechanism known as nonsense-mediated mRNA decay (NMD) that degrades the PTC-containing mRNA. The activation of NMD can attenuate the consequences of truncated, defective, and potentially toxic proteins in the cell. Since approximately 20% of all single-point mutations are disease-causing nonsense mutations, it is not surprising that this field has received significant attention, resulting in a remarkable advancement in recent years. In fact, since our last review on this topic, new examples of nonsense suppression approaches have been reported, namely new ways of promoting the translational readthrough of PTCs or inhibiting the NMD pathway. With this review, we update the state-of-the-art technologies in nonsense suppression, focusing on novel modalities with therapeutic potential, such as small molecules (readthrough agents, NMD inhibitors, and molecular glue degraders); antisense oligonucleotides; tRNA suppressors; ADAR-mediated RNA editing; targeted pseudouridylation; and gene/base editing. While these various modalities have significantly advanced in their development stage since our last review, each has advantages (e.g., ease of delivery and specificity) and disadvantages (manufacturing complexity and off-target effect potential), which we discuss here.

## 1. Introduction

Premature termination codons (PTCs) occur when mutations change a sense codon into an early stop (nonsense) codon. The ribosomal machinery can recognize a PTC as an early termination signal for the translation process. To prevent the accumulation of a potentially toxic truncated protein in the cell, the nonsense-mediated mRNA decay (NMD) surveillance pathway is activated, degrading PTC-containing mRNA transcripts. A small fraction of the mRNA transcripts evades NMD and are translated, but the translation terminates at the PTC, generating a C-terminally truncated protein. The lack of a full-length functional protein can cause disease, also known as nonsense-related disorder. Many human genetic diseases, such as cystic fibrosis [1], neurofibromatosis type 1 [2], beta-thalassemia [3], inherited bone marrow failure syndromes [4], and Duchenne muscular dystrophy [5], can have this same root cause. A potential way to treat these diseases while addressing the underlying cause would be to employ a therapy that would repair the nonsense mutation at the mRNA level. Such an approach designed to correct nonsense codons would enable the ribosomal machinery to “readthrough” the PTC until it meets the normal stop codon downstream, thus restoring full-length protein production. It is desirable that this approach would also suppress NMD, allowing the accumulation of the repaired mRNA for normal translation. There are small molecules capable of promoting translational readthrough at the PTC. However, the lack of target specificity is one of the main challenges. In other words, it is difficult to direct the small molecules, which often bind/target the ribosomes, to act only at the PTC sites and not at normal stop codons. In recent years, many laboratories have developed nucleic acid-based technologies to address nonsense mutations, taking advantage of the specificity of Watson–Crick base pairing rules. In a previous paper [6], we summarized different nonsense suppression strategies powered by different technologies, especially emerging ones. Here, we describe the recent advances in many of those emerging technologies, explaining the fundamentals of each modality (i.e., their molecular mechanism of action), emphasizing medical applications, and the opportunities they can offer for personalized medicine.

## 2. Translation Termination

**Figure 1:**
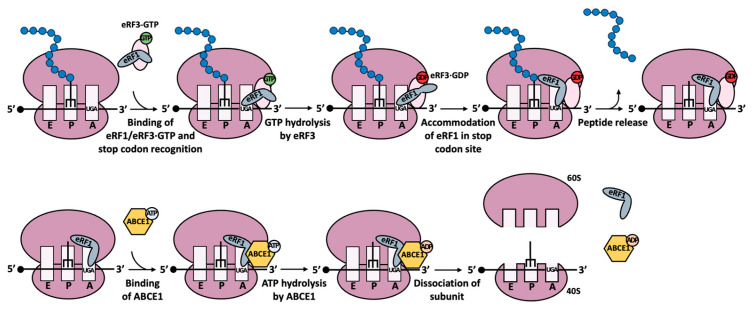
Mechanisms of translation termination. When the ribosome encounters a stop codon (e.g., UGA), a ternary complex consisting of eukaryotic release factor 1 (eRF1), eukaryotic release factor 3 (eRF3), and guanosine triphosphate (GTP) (also known as the eRF1/eRF3-GTP complex), rather than a tRNA, recognizes it and binds to the A site. In the next step, eRF3 hydrolyzes GTP, and subsequently, eRF1 accommodates at the stop codon site while promoting the release of the peptide chain. Then, the ATP-binding cassette subfamily E member 1 (ABCE1) binds to the post-termination ribosome complex and hydrolyses ATP, thus promoting the dissociation of the 80S ribosome.

Translation termination is a crucial stage of protein synthesis. The ribosomes move along the mRNA during the translation elongation phase, successively adding amino acids into a growing polypeptide chain. This translation process relies on the recognition of each codon by the corresponding transfer RNA (tRNA). Specifically, the anticodon of the tRNA base pairs with the corresponding codon, ensuring that the correct amino acid is incorporated during translation. However, when a ribosome encounters a stop codon (UAG, UAA, or UGA), no tRNA recognizes it. Instead, the release factors enter the ribosome to recognize the stop codon, thereby terminating protein translation via a peptidyl-tRNA hydrolysis reaction and promoting the release of the growing polypeptide chain. In eukaryotes, this release factor is known as eukaryotic translation termination factor 1 (eRF1, a class 1 release factor), which can recognize the three different stop codons [7,8]. Eukaryotic translation termination factor 3 (eRF3) is a GTPase that can form a complex with eRF1 and guanosine triphosphate (GTP), thus contributing to translation termination [7,9]. This ternary complex [9,10,11] binds to the stop codon at the A site, mimicking a tRNA-like structure. Subsequently, eRF3 hydrolyzes GTP and dissociates from the complex, while eRF1 promotes the release of the terminated peptide chain via hydrolysis of the peptidyl-tRNA ester bond. In the next step, ABCE1, an ATP-binding cassette protein, binds to the post-termination complex and hydrolyzes ATP, leading to the split of the 80S ribosome and the release of the 40S and 60S subunits (Figure 1) [12].

The translation termination mechanism at normal or premature stop codons is not 100% efficient. It has been estimated that up to 0.1% of basal (i.e., natural) stop codon readthrough can be achieved through the recognition of the stop codon by a near-cognate tRNA instead of eRF1 [13]. On the other hand, the basal premature stop codon-readthrough could be as high as 1% [14]. The exact frequency of these events could depend on various factors: the identity of the stop codons, their surrounding sequence context, or any potential secondary structures occurring in the 3′-untranslated region (UTR) [15]. Recently, it has been suggested that the tRNA abundance could also play a potential role [16].

## 3. Nonsense-Mediated mRNA Decay Surveillance Pathway

When a ribosome encounters a PTC, a cellular surveillance pathway, called the nonsense mRNA-mediated decay (NMD) pathway, is activated to degrade the PTC-harboring transcripts. NMD is an essential and highly conserved cellular mRNA quality control mechanism responsible for the degradation of aberrant transcripts (e.g., PTC-containing mRNAs). This pathway also appears responsible for controlling the abundance of other mRNAs that do not harbor a PTC [17].

Usually, the NMD mechanism degrades most PTC-containing transcripts, thereby preventing the synthesis of truncated and potentially toxic proteins. However, a small fraction of the mRNA transcripts escape degradation and are translated into the C-terminally truncated protein. Usually, the proteasome machinery degrades the defective protein; however, when that is not possible, aggresomes can accumulate until they are degraded via the autophagic pathway. This accumulation of the truncated peptides depends on hyperphosphorylated UPF1, a key component of the NMD surveillance pathway [18].

**Figure 2:**
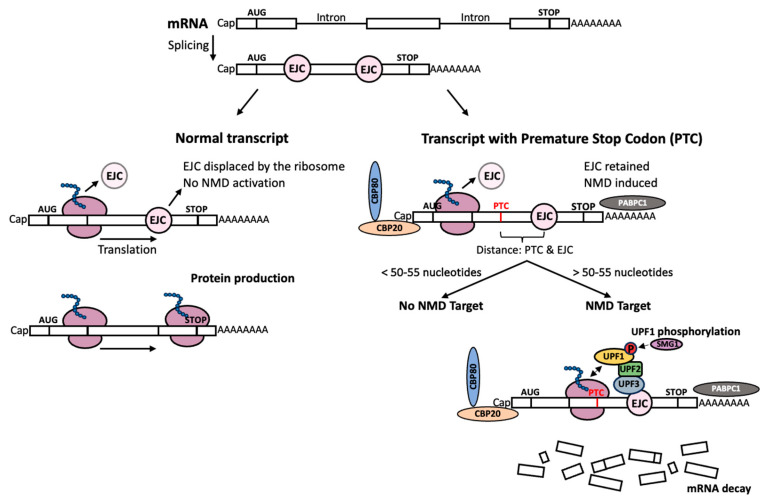
Mechanisms of the nonsense-mediated mRNA decay (NMD). During splicing, exon junction complexes (EJC) assemble at exon–exon junctions in the mature mRNA. These multiprotein complexes are usually displaced by the ribosome in transcripts containing only the normal stop codon. The nonsense mRNA-mediated decay (NMD) pathway is not activated in this case. On the other hand, when a premature stop codon (PTC) is present in the transcript at a distance greater than 50–55 nucleotides from the closest EJC, the mRNA becomes a target for degradation via the NMD mechanism. Proteins such as UPF1, UPF2, and UPF3 interact with each other and the EJC, while the ribosome stalls at the PTC site. Then, the UPF1 phosphorylation mediated by SMG1 precedes the exonucleolytic degradation of the PTC-harboring mRNA.

NMD is present in all eukaryotes, and its mechanism of action is evolutionarily conserved. During pre-mRNA splicing, a multiprotein complex, called the exon junction complex (EJC), is deposited at each exon–exon junction. Normally, all the EJCs are displaced by the moving ribosome during the pioneer round of translation. However, when a nonsense mutation occurs more than 50–55 nt upstream of the nearest exon–exon junction (also known as the “50–55 nt rule”), NMD is triggered (Figure 2) [19]. Specifically, several proteins in the EJC, such as UPF2 and UPF3B (also Y14, MAGOH, elF4A3, MLN51, and RBM8A), form crucial interactions with each other. On the other hand, the translating ribosomes stall when encountering the PTC, prompting the eRF1–eRF3 complex to recruit UPF1, SMG1, SMG8, and SMG9 to form the SURF complex [20]. Subsequently, the decay-inducing (DECID) complex forms (a combination of both the SURF and EJC complexes), triggering the phosphorylation of UPF1, the ultimate repression of translation, and the exonucleolytic degradation of the targeted mRNA (Figure 2) [17]. Since the EJC complex forms during splicing (before the mRNA is exported to the cytoplasm), intronless genes are usually NMD-insensitive [21].

A better understanding of the NMD pathway is crucial for accelerating the development of safer and more effective therapeutic readthrough strategies, as NMD inhibition could leverage translational readthrough effects. In this context, there is a growing need for animal models to understand the consequences of NMD inhibition better. Due to the importance of this pathway to the cell, some attempts were made to generate in vivo models where the knockdown of crucial NMD-related genes somewhat suppressed NMD. However, such approaches led to embryonic mortality [22], a concern for NMD inhibition strategies.

A few years ago, Echols et al. developed a potentially valuable NMD mouse model to understand the consequences of NMD inhibition at different levels in different aspects of animal development [23]. In this mouse model, there is an inducible dominant-negative form of human UPF1, thus allowing the prediction of the impact of NMD inhibition at different levels and in different mouse tissues. By inducing the level of NMD inhibition, the authors could avoid the developmental problems that could have originated from a complete knockdown of a crucial NMD factor, such as UPF1 [22]. The data suggest that strategies aimed at inhibiting UPF1, particularly if capable of achieving high inhibition levels, could lead to adverse effects and need proper safety assessments in additional preclinical models.

## 4. Nonsense Suppression Strategies

**Figure 3:**
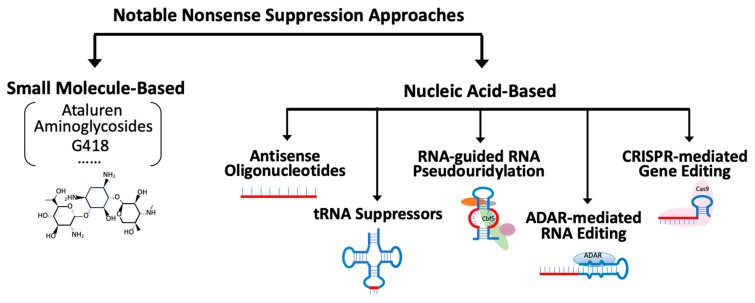
Some notable nonsense suppression strategies include small molecule-based and nucleic acid-based approaches. Several small molecule classes have been described with readthrough properties, such as oxadiazoles (e.g., Ataluren) or aminoglycosides. On the other hand, nucleic acid-based approaches, such as RNA editing, targeted pseudouridylation, or gene editing, take advantage of hybridization properties to specifically target transcripts (or genes) containing a premature stop codon. Abbreviations: ADAR: adenosine deaminases acting on RNA; CRISPR: clustered regularly interspaced short palindromic repeats.

There are at least two notable nonsense suppression strategies: small molecule-based and nucleic acid-based modalities (Figure 3). Each has advantages and disadvantages. Several approaches are being developed in academic groups and biotech/biopharma companies.

### 4.1. Small Molecules

#### 4.1.1. Small Molecules That Promote PTC Readthrough

Compared to more complex modalities, particularly nucleic acid-based ones (discussed below), small molecules have several advantages in drug development. For instance, small molecules are known to be capable of penetrating tissue and cell membranes. They are also likely to be administered orally due to their absorption properties [24]. Further, small molecules can be theoretically more affordable than complex RNA-based or gene and cell therapies. Therefore, increasing efforts have been made in recent years to develop small molecules as readthrough agents [25]. A well-known class of small molecules that promote translational readthrough are aminoglycosides [26], which have historically been used as antibiotics [27]. Ataluren, an oxadiazole discovered to suppress Duchenne muscular dystrophy nonsense mutations, is also renowned [28]. The mechanisms of action of these nonsense suppressor molecules have been described to some extent [29,30]. For example, Ng et al. proposed PTC readthrough orthogonal mechanisms for both ataluren and G418. Ataluren interferes with the translation termination process by inhibiting the activity of a release factor, whereas G418 promotes readthrough via a mispairing interaction between the PTC mRNA and the near-cognate tRNA [31].

Despite the challenges, small molecules have a promising clinical potential in promoting PTC readthrough. Moreover, they have a crucial role as positive controls and tool compounds for preclinical validation of the suppression of newly discovered nonsense mutations [32,33] or for the validation of novel advanced preclinical in vitro and in vivo models [34,35].

#### 4.1.2. Small Molecules That Suppress NMD

The use of small molecules to inhibit the NMD pathway has also attracted interest. NMD inhibition could have an additive effect when combined with readthrough agents, leading to an increased production of full-length protein and phenotypical reversal [36]. One of the early examples published of a NMD inhibitor small molecule was NMDI 1 (or NMD inhibitor 1) [37], which could prevent the interaction between two proteins that play a role in the overall NMD pathway, human UPF1 [38] and SMG5 [39]. However, the authors of this study acknowledged the risk for the preclinical and clinical development of NMDI 1, given that it is an indole derivative and a new chemical class. To lower the risk, the screening of a library of marketed drugs was also carried out, leading to the identification of amlexanox, a well-known immunomodulator drug (also known as Aphthasol^®^, used in the treatment of aphthous ulcers) that was found to inhibit the NMD pathway [40].

More recently, another screening approach was carried out using currently FDA-approved small molecules [41]. The researchers were able to identify an anticancer drug (for the treatment of chronic myeloid leukemia), homoharringtonine (or HHT), which was capable of inhibiting NMD in specific mRNA substrates such as *Ptbp2*, *Hnrnpl*, and *Tra2b*, starting from a concentration of 50 nM, in a dose-dependent manner in human cells.

#### 4.1.3. Combined Effect of NMD Inhibitors and PTC Readthrough Agents

With the availability of NMD inhibitors (small molecules) and PTC readthrough agents (small molecules), research groups are finally exploring their combined effect on nonsense suppression in several in vitro and in vivo model systems.

McHugh et al., for example, analyzed the combined effect of both approaches in a CF mouse model carrying the G542X nonsense mutation in the *CFTR* gene. The authors found that inhibiting SMG1 while promoting readthrough with G418, gentamicin, or paromomycin could benefit the overall nonsense suppression effect [36]. Similarly, Wang et al. found that amlexanox could be combined with ataluren to promote phenotypical reversal (improved vision and reduced death of retina cells) in a zebrafish model of Leber’s congenital amaurosis (LCA) expressing a nonsense mutation in the *CEP290* gene (Q1223X) [42]. When attempting to replicate these results in mammalian LCA mouse models, the authors found that some nonsense mutations could be better suppressed than others.

It should be noted that inhibiting NMD in a non-sequence/gene (or target)-specific manner would be challenging, considering how ubiquitous this pathway is for cellular homeostasis and the regulation of multiple genes. The inhibition of NMD components such as SMG1 has been shown to potentially lead to toxic effects, especially if combined with high doses of readthrough agents such as aminoglycosides [36].

#### 4.1.4. Development of New Nonsense Suppression Small Molecules

There have been developments in new synthetic aminoglycosides as readthrough agents. One of them is NB124 (or ELX-02), which initially showed promising results in restoring the function of the cystic fibrosis transmembrane conductance regulator (CFTR) channel in a mouse model [43]. Following the initial clinical tests in healthy patients [44] where the compound showed tolerability and a safety profile with no severe or significant drug-related side effects, the molecule was tested in a Phase 2 clinical trial for efficacy in patients either homozygotes for G542X or compound heterozygotes with one G542X or a phenotypically similar nonsense allele and any class I or II mutation). Unfortunately, ELX-02 was ineffective in CF patients, possibly due to the insufficient exposure of ELX-02 in the lung tissue [45].

Two additional potential readthrough compounds were also recently discovered. One is 2-guanidino-quinazoline (TLN468), discovered using a reporter cell line where the most common DMD PTC sequence contexts could be tested [46]. Another example is 2,6-diaminopurine, shown in a study to have potential therapeutic use in the readthrough of *CFTR* nonsense mutations, specifically as a readthrough agent of UGA nonsense mutations in this gene, such as G542X [47]. The amino acid incorporated at the UAG PTC position was tryptophan. It has been proposed that the readthrough mechanism of 2,6-diaminopurine could rely on disturbing the activity of a tRNA^Trp^-modifying enzyme (FTSJ1) [48]. The 2,6-diaminopurine compound was shown to be capable of restoring the function of the CFTR channel in a CF mouse model carrying a *CFTR* nonsense mutation [47].

#### 4.1.5. Small Molecules with Novel Mechanisms

Novel small molecule modalities for nonsense suppression are still being explored for undruggable targets. A promising example is the use of a molecular glue degrader. Coelho et al. recently proposed using an eRF1 degrader small molecule, “SRI-41315”, which could promote translational readthrough of premature stop codons [49]. The authors of this study performed Cryo-EM to show that SRI-41315 could work as a metal-dependent molecular glue between the region near the decoding site of the ribosomal subunit and eRF1 (at its stop codon recognition N domain). Sharma et al. have already shown that SRI-41315 depletes eRF1 in the cell, thus promoting the suppression of *CFTR* nonsense mutations [50]. Another example of molecular degraders with PTC readthrough activity was presented by Baradaran-Heravi et al. [51]. Here, the authors identified two molecular glue compounds, CC-885 and CC-90009, capable of promoting the proteosome-mediated degradation of crucial translation termination eukaryotic release factors eRF3a and eRF3b, both of which are involved in stop codon recognition through interaction with eRF1 [52].

### 4.2. Nucleic Acid-Based Approaches

New developments in modalities based on much larger molecules have been reported in recent years. These modalities are primarily based on nucleic acids (e.g., nuclease-encoded mRNA, guide RNAs, antisense oligonucleotides, tRNAs, and H/ACA snoRNAs). Although more complex than small molecule nonsense suppression agents, nucleic acids offer better target specificity and flexibility through platform technologies (e.g., with modified guide RNAs), which can be engineered to address virtually any nonsense mutation, irrespective of the sequence context. Here, we discuss several promising approaches.

#### 4.2.1. Antisense Oligonucleotides

Oligonucleotides are among the most important emerging modalities in the biotech and biopharma industries. Oligonucleotides can potentially repair nonsense mutations in several different ways, which we discuss in this section. Antisense oligonucleotide (ASO)-directed exon skipping was one of the first oligo-mediated approaches described decades ago. In this approach, an ASO is designed to target the PTC-containing exon, promoting the skipping of it [53,54] while maintaining the function of the PTC–exon-deleted protein [55]. However, it should be pointed out that, in some cases where a mutation leads to aberrant splicing that generates a pseudo-exon with a PTC [56], the ASO-mediated skipping of this PTC-containing pseudo-exon can restore the wild-type mRNA and protein sequences [55,57]. Another ASO-based approach uses gapmers (i.e., RNase H-recruiting antisense oligonucleotides) designed to trigger the degradation of mRNA transcripts encoding important NMD components, leading to the maximization of the level of PTC-containing mRNA for translation [58,59,60]. Alternatively, the gapmers are also used to target the degradation of translation termination components, thus maximizing PTC readthrough [61]. Finally, a third strategy to tackle nonsense mutations with ASO involves designing steric-blocking oligonucleotides that can bind to a sequence downstream of a PTC in order to prevent EJC deposition, which is crucial for NMD activation; preventing EJC deposition would inhibit NMD [62,63].

#### 4.2.2. tRNA Suppressors

The suppression of nonsense mutations can be achieved by artificial tRNAs designed to recognize PTCs via codon–anticodon base pairing. This concept was proposed decades ago as a gene therapy approach for nonsense suppression [64]. These artificial tRNAs are also known as tRNA suppressors. tRNA suppressors are engineered to be recognized by the translational machinery and to deliver an amino acid at the PTC during translation. Because its anticodon is altered to form base pairing interactions with the PTC, a tRNA suppressor, upon charging, enters the ribosome to read the PTC as if it were a normal codon, thus enabling translational readthrough [65,66]. As a gene therapy, this modality could potentially be delivered either encapsulated in lipid nanoparticles [67] or encoded into recombinant adeno-associated virus (rAAV) via a tRNA transgene [68]. Perhaps for this reason, this emerging modality has attracted attention [69], with several biotech companies exploring this space (e.g., Recode Therapeutics, AlltRNA, hC Bioscience, and others) [70]. A comprehensive review was recently published describing the history of tRNA suppressors, design considerations, delivery approaches, and overall challenges in this field [71].

Suppressor tRNAs can be screened using high-throughput assays to select the best-engineered sequences for nonsense suppression in vitro and in vivo [72]. This approach was used, for example, to suppress *CFTR* nonsense mutations in an endogenous (sequence) context using Clustered Regularly Interspaced Short Palindromic Repeats (CRISPR) gene-edited human bronchial epithelial cells (also known as 16HBEge cells) [73,74]. Engineered suppressor tRNAs designed to target different *CFTR* nonsense mutations [G542X (UGA), R1162X (UGA), and W1282X (UGA)] were able to rescue the protein function and restore the mRNA levels. The authors of this study also suggested that this approach could inhibit the NMD pathway in the first round of mRNA translation. Further, tRNA suppressors have been recently successfully delivered to animals either by being encoded in rAAVs (transducing in the heart, brain, liver, and skeletal muscles) [68] or via their encapsulation in LNPs (targeting liver and lung tissues) [67].

Similarly to other nonsense suppression modalities, the efficiency of tRNA suppression must be somewhat dependent on the sequence context of PTC. Recently, Bharti et al. compared the efficiency of tRNA suppressors in different PTC contexts [75]. The authors found that the translation speed of ribosomes before they reach the PTC could impact the tRNA suppression effect. If a specific sequence context upstream of the PTC site provides a sudden reversal in translation speed (resulting in more ribosome collisions), tRNA suppression could be hampered.

#### 4.2.3. ADAR-Mediated RNA Editing

Guide RNAs can be designed to direct specialized enzymes (known as adenosine deaminase acting on RNA, or ADAR) in human cells to catalyze RNA editing, converting specific adenosines into inosines. Inosines can be read as guanosines by the translational machinery. While RNA editing is a naturally occurring mechanism targeting endogenous RNAs, it can also be directed using guide antisense RNAs [76]. This mechanism of action can potentially be used to repair nonsense mutations, since all premature stop codons have at least one adenosine nucleotide (UGA, UAG, and UAA). For example, artificial guide RNA-directed ADAR editing of adenosine(s) to inosine(s) in PTCs would enable to convert a PTC (UAG, UAA, or UGA) into a tryptophan codon (UGG). For nonsense mutations resulting in a conversion of a tryptophan sense codon into a PTC (i.e., W-to-X), this would change the PTC back to the tryptophan codon (wild-type), a best-case outcome in a therapeutic context.

Recently, multiple groups have suggested employing guide RNAs that utilize either internally expressed or externally delivered ADAR machinery (in cis or trans) for RNA editing in human cells [77,78,79,80,81,82]. The guide RNAs can be chemically modified oligonucleotides [82,83,84,85] or rAAV-delivered guide RNAs [86] or even circular RNAs [87].

Different research groups have proposed screening assays to identify the best guide RNA sequences for A-to-I editing at PTCs. For instance, Diaz Quiroz et al. developed an in vitro assay to select optimized guide RNAs to target an adenosine residue within a PTC [88]. On the other hand, Schneider et al. presented a yeast system to identify the best ADAR targets that are more suitable for A-to-I editing [89]. The authors also discovered the most efficient guide RNAs to edit retinal disease targets carrying nonsense mutations using this assay.

Since the first in vitro proof-of-concept studies showed the feasibility of ADAR-mediated nonsense suppression, further experiments have been carried out, demonstrating this approach in small animals as well [86,87,90,91]. Although not targeting endogenous PTCs, ADAR-mediated RNA editing has also been demonstrated in non-human primates using rAAV-encoded guide RNAs [92] or stereo-pure chemically modified oligonucleotides [84]. The first clinical trial using RNA editing oligonucleotides has recently been initiated for treating Alpha-1 antitrypsin disease (AATD). It is not aimed, however, at suppressing a nonsense mutation but, rather, a missense correction (E342K) in the *SERPINA1* transcript [93]. Nevertheless, a biotech company has a preclinical program for Rett syndrome, where RNA editing targets a well-known nonsense mutation (*Mecp2*-R255X) [94].

As for the other modalities described in this review, implementing ADAR-mediated RNA editing as a nonsense suppression could be challenging. For instance, when taking advantage of an endogenously expressed ADAR enzyme, it needs to be taken into account that the expression of ADAR varies not only in different tissues [95] but also in different species [96]. When targeting tissues with poor ADAR expression, the delivery of exogenously expressed ADAR could be necessary. However, this could bring challenges such as immunogenicity and off-target effects [97,98].

Finally, it is still unclear whether RNA editing affects the NMD pathway. It appears that RNA editing could have an impact on splicing, as demonstrated by Hsiao et al., who showed that more than 95% of A-to-I RNA editing occurred co-transcriptionally in nascent RNAs when they were still associated with chromatin and before poly-adenylation [99]. Along the same line, another study found that the A-to-I conversion can be more efficient at a pre-mRNA’s PTC than at a mature mRNA’s PTC [100]. While these results suggest that RNA editing affects splicing, in a therapeutic context, it remains to be investigated which ADAR isoform (cytoplasmic versus nuclear-localized) is recruited by exogenous PTC-targeting guide RNAs. This could be important when not delivering an exogenous ADAR, especially considering that the ADAR1 p150 isoform is mainly localized in the cytoplasm, while ADAR1 p110 is primarily found in the nucleus [101].

#### 4.2.4. Targeted RNA Pseudouridylation

Pseudouridine (Ψ), an isomer of uridine, is one of the most abundant modified nucleotides in RNAs. Because of the successful use and critical role of a Ψ derivative in approved effective COVID-19 mRNA vaccines, Ψ has recently attracted significant attention [102]. Remarkably, pseudouridylation can be catalyzed by an RNA-guided mechanism discovered nearly 30 years ago when a large family of box H/ACA RNAs was identified in cells [103,104,105]. Despite the sequence differences, the box H/ACA RNAs fold into an identical structure, which complexes with four core proteins (Cbf5, Nhp2, Nop10, and Gar1) to form a ribonucleoprotein (RNP) [103,104,105,106,107]. During modification, the RNA component (box H/ACA RNA) of the RNP serves as a guide that pairs bases with the substrate RNA, precisely positioning the target uridine in the catalytic center of the pseudouridylase Cbf5 to be pseudouridylated [103,105,108]. The site-specific nature of this RNA-guided process is experimentally verified [103,105,108,109]. As expected, by changing the guide sequence of a naturally occurring box H/ACA RNA, Karijolich et al. succeeded in redirecting pseudouridylation to new RNA sites that are otherwise unmodified [110]. One such specific target site was the PTC of a mRNA. Remarkably, target U-to-Ψ conversion at the PTC led to NMD suppression and PTC readthrough [110,111,112]. It was also reported that pseudouridylation occurred naturally at some normal stop codons, promoting stop codon readthrough and the production of C-terminally extended proteins [113]. These results have opened the door to the possibility of developing the RNA-guided PTC pseudouridylation technology into therapeutic drugs for diseases caused by nonsense mutations.

Recently, the RNA-guided RNA pseudouridylation technology was further tested to suppress nonsense mutations in various sequence contexts, many of which were derived directly from patients [112,114]. For instance, it has been reported that targeted pseudouridylation converting UAA, UAG, or UGA to ΨAA, ΨAG, or ΨGA, respectively, in the context of disease genes, including *β-globin* (*β*-thalassemia), *CFTR* (cystic fibrosis), *IDUA* (Hurler syndrome), *TP53* (cancer), and *NF1* (neurofibromatosis type 1), resulted in both NMD suppression and PTC readthrough [114]. The Ψ-mediated nonsense suppression appears to be sequence context-independent [115]. However, the PTC sequence must be accessible and available for the guide RNA to pair bases with it. Although the efficiency of nonsense suppression is still relatively low, overexpression of relevant tRNAs has dramatically improved nonsense suppression [116], suggesting that this technology has great potential in clinical applications.

#### 4.2.5. CRISPR-Mediated Gene Editing

Since CRISPR technology was discovered, it quickly became a valuable tool to generate advanced preclinical models with therapeutically relevant mutations, particularly nonsense mutations, such as, for instance, isogenic cell lines [74], organoids [117], or animal models [34,118]. In recent years, examples of how CRISPR technology and its variations could potentially be used in the context of nonsense suppression have been reported [119]. By correcting a nonsense mutation at the DNA level with a nuclease of bacterial origin (e.g., Cas9) and a guide RNA matching the target site, CRISPR gene editing circumvents the abovementioned issues with the NMD pathway. However, traditional CRISPR-Cas9 generates a break in the DNA. Alternatively, base editing can repair mutations at the nucleobase level without performing a DNA double-stranded break [120]. Base editors, instead of an active nuclease, have a deactivated nuclease (e.g., dCas9) fused to an engineered base deaminase [121,122], which performs editing of certain nucleobases (e.g., the deamination of cytosines into uridines or the deamination of adenosines into inosines) in a programmed manner, thanks to single guide RNA (sgRNA). An example of the base editing approach for correcting nonsense mutations in vivo was recently published. In this study, the components for the adenosine base editor (ABE) system were split into a dual AAV9, which was delivered either locally (intramuscular injection) or systemically (intraperitoneal injection) to correct Duchenne muscular dystrophy (DMD) nonsense mutations in a DMD mouse [123].

The first CRISPR-based gene therapy was recently approved [124,125]. While it is still an ex vivo treatment for blood disorders (and not an in vivo systemically delivered drug), this modality has enormous potential, as it could cure nonsense disorders with a single treatment. Still, some challenges remain for gene and base editing, namely off-target effects [126] and potential immunogenicity elicited by the exogenous editing machinery [127].

## 5. Conclusions

Since our last review on this subject [6], new advances in nonsense suppression modalities have been described, both in the small molecule space and nucleic acid-based approaches. The different modalities have intrinsic advantages and disadvantages (Table 1).

The development of small molecule nonsense suppression agents holds great promise for treating nonsense disorders. Despite promising preclinical studies, clinical success is still difficult to achieve. There are multiple ways in which small molecules can somehow promote readthrough and/or inhibit the NMD pathway. Many researchers in this space have evaluated for several years which pathways show the most promise in developing clinically effective nonsense suppression agents. Perhaps more answers could emerge from yet-to-be-discovered pathways or combined therapies targeting several nonsense-related mechanisms concurrently. More therapeutic interventions could also arise from a combination of nonsense suppression agents with corrector molecules [128,129] for the instances where the incorporated amino acid does not fully restore the protein’s function. Further, a proper pharmacokinetic and pharmacodynamic profiling of readthrough agents in preclinical studies is essential for creating treatments that achieve sufficient tissue exposure [45]. A recent review discussed important preclinical aspects that need further consideration to develop safe and effective small molecule readthrough therapies [25].

On the other hand, nucleic acid-based modalities offer a plethora of mechanisms of action with different complexities to correct or repair the underlying cause of nonsense disorders. Since our last review discussing nucleic acid-based strategies [6], there have been multiple breakthroughs in several enabling technologies, such as chemical modification patterns [130], manufacturing methods [131], tissue-specific oligonucleotide conjugates for targeted delivery [132], and innovative viral or non-viral delivery methods [133,134]. Arguably, the wealth of knowledge from dozens of clinical trials establishing new regulatory pathways for future developments is almost as significant. This is an essential aspect, particularly in the context of new modalities.

However, despite the technological developments described in this review, it is still necessary to further understand the biology of nonsense suppression in order to develop safer and more efficacious readthrough drugs. For instance, in the context of the development of nonsense suppression strategies, it is essential to understand the exact role of NMD in the PTC transcript of interest. The NMD efficiency could impact the disease phenotype, for example, if a particular readthrough approach is less effective, it would be crucial to increase the mRNA level to boost protein translation to a level that is potentially above the threshold needed to reduce phenotype severity. It is also worth considering that readthrough efficiencies are often calculated in many studies using overexpressed fluorescent intron-lacking reporter systems [135], which could have some limitations in fully representing the endogenous expression of PTC-containing transcripts in primary cells.

## Figures and Tables

**Table 1:** State-of-the-art therapeutic nonsense suppression modalities: mode of action, molecular targets, potential advantages, and current challenges of the different approaches discussed in this review. Abbreviations: NMD: nonsense-mediated mRNA decay; PTC: premature stop codons; ADAR: adenosine deaminases acting on RNA; LNP: lipid nanoparticles; AAV: adeno-associated virus.

Modality	Mode of Action	Targets	Advantages	Challenges
**Small Molecules**	Readthrough promoters, NMD inhibition [29,30,36]	Ribosomal machinery, translation termination factors, NMD factors [36,48,49]	Cellular penetration Oral administration Ease of manufacturing Compound combination (readthrough and NMD inhibition) [24,25]	Target specificity Variable efficiency in different sequence contexts Need for frequent administration
**Nucleic Acid-based Approaches**	Antisense Oligonucleotides	Skipping of PTC-harboring transcripts, degradation of NMD or translation termination component [53,54,55,56,57,58,59,60,61,62,63]	Clinically validated platform Target specificity Duration of effect Enabling platforms	No PTC repair PTC-exon skipped transcript might not be functional Toxicity when targeting NMD or translation termination factors
	tRNA Suppressors	PTCs (mRNA) via codon-anticodon base pairing [65,66]	Delivered in LNPs or as gene therapy [67,68]	Off-target effects Efficiency dependent on sequence context [71]
	ADAR-mediated RNA editing	PTCs (mRNA) via A-to-I [76,77,78,79,80,81,82]	Delivered as modified oligos or AAV-encoded guide RNAs Potential for reversal to wild-type (W-to-X nonsense mutations) [76,82,83,84,85,86,87,92]	Expression of ADAR in different tissues Unclear if affects NMD Immunogenicity (if ADAR is co-delivered) [95,96,97,98,99,100]
	Targeted RNA Pseudouridylation	PTCs (mRNA) via U-to-Ψ [110,111,112]	Repair in different sequence contexts Endogenous machinery ubiquitously expressed NMD inhibition [112,113,114]	Efficiency (can be boosted by co-expression of a tRNA) [116]
	CRISPR-mediated Base Editing	PTCs (DNA)	No DNA double-stranded break required Single treatment [120,121,122]	Permanent off-target effects Immunogenicity (exogenous machinery) [126,127]
